# RNA polymerase I-driven reverse genetics system for enterovirus 71 and its implications for vaccine production

**DOI:** 10.1186/1743-422X-9-238

**Published:** 2012-10-17

**Authors:** Tao Meng, Tanja K Kiener, Jimmy Kwang

**Affiliations:** 1 Animal Health Biotechnology, Temasek Life Sciences Laboratory, National University of Singapore, 1 Research Link, Singapore, 117604, Republic of Singapore; 2 Department of Microbiology, Yong Loo Lin School of Medicine, National University of Singapore, Singapore, Republic of Singapore

**Keywords:** Enterovirus 71, Universal RT-PCR, Reverse genetics, Vaccine

## Abstract

**Background:**

Enterovirus 71 (EV71) is a virus that causes from mild hand, foot and mouth disease (HFMD) to severe neurological complications and deaths in infants and young children. Effective antiviral agents and vaccines against EV71 are not available. However, Vero cell-based chemically inactivated EV71 vaccines could be developed soon based on the success of inactivated polio vaccine. Like poliovirus, EV71 has a positive single-stranded RNA genome of about 7400 nucleotides which contains a single open reading frame (ORF) flanked by conserved and untranslated regions at both the 5^′^ and 3^′^ ends.

**Results:**

The universal amplification of the full length genome of EV71 regardless of its genetic diversity, and the subsequent construction of a human RNA polymerase I-driven reverse genetics (RG) system to produce pure virus stocks in Vero cell within 10 days were described. The rescued viruses were characterized by DNA sequencing, cytopathic effect (CPE) and indirect fluorescent assay (IFA) in comparison with the wild-type viruses. Moreover, the rescued viruses grew to high titers and retained the same immunogenicity as the wild-type viruses.

**Conclusion:**

We have established a simplified method to rescue RG EV71 virus from diverse clinical isolates with detailed genetic information and to prepare virus stocks in only 10 days. This method could accelerate EV71 vaccine development.

## Background

Enterovirus 71 (EV71) belongs to the enterovirus genus of the picornavirus family, which also includes coxsackievirus A16 (CA16), poliovirus and echovirus [1]. EV71 has a small and nonenveloped icosahedral capsid comprising of four structural proteins (VP1 to VP4) and surrounding a genome of ~7,400 nt single stranded RNA. The viral genome contains a single large open reading frame (ORF) encoding a polyprotein which is subsequently cleaved into multiple mature proteins by virally encoded proteases. The ORF is flanked by 5^′^- and 3^′^- untranslated regions (5^′^- and 3^′^- UTR) which are highly conserved and play essential roles during the viral life cycle. There is also a short poly A tail following the 3^′^-UTR [2,3]. The EV71 genome mutates rapidly because of the low fidelity of its RNA dependent RNA polymerase [4,5]. Based on a phylogenetic tree of VP1 gene sequences, EV71 is divided into three major genogroups (denoted A, B and C), and various subgenogroups within genogroups B (B1 to B5) and C (C1 to C5) [4-6]. Predominant subgenotypes currently circulating are B5, C1, C4 and C5; and different genotypes of EV71 strains may co-circulate in the same area. Additionally, intra- or inter-genogroup recombination and positive selection contribute to the genetic and antigenic diversity of EV71 [7,8].

EV71 is a causative agent of hand, foot and mouth disease (HFMD), most frequently affecting infants and children below 6 years old. Infection with EV71 is usually mild but occasionally leads to neurological manifestations ranging from aseptic meningitis to acute flaccid paralysis and lethal brainstem encephalitis [9,10]. Large outbreaks of EV71 with fatal cases have been seen in the Asia-Pacific region since the 1990s [2,6,11]. Unfortunately, no effective medications or prophylactic vaccines are currently available for controlling EV71 infection. Mass vaccination of formaldehyde inactivated or adapted live attenuated EV71, grown in African green monkey kidney (Vero) cell, would be a favorable method to control EV71 epidemics based on the successful experience in poliovirus vaccination [12]. Therefore, the selection and characterization of EV71 vaccine strains with high growth titers and broad cross-subgenogroup virus neutralizing antibody responses are urgent for vaccine development [13].

Reverse genetics (RG) permits the use of complementary DNA (cDNA) copies of viral RNA genome to rescue virus with detailed features of viral genetic diversity, antigenicity and virulence. RG systems using T7 or S6 RNA polymerase to synthesize infectious RNA *in vitro* from cDNA have been developed for many picornaviruses, including EV71 [14,15]. In addition, RNA polymerase I-based RG systems which ensure the precise and efficient transcription of viral genomes *in vivo* have been described for influenza virus [16], HFMD [17] and EV71 [18].

Here, we have developed a robust RT-PCR method for the universal amplification of whole EV71 genome and a novel human RNA polymerase I (hPolI)-driven RG system for the rapid production of pure and immunogenic stock in Vero cell. The implications of these findings are discussed.

## Results

**Figure 1:**
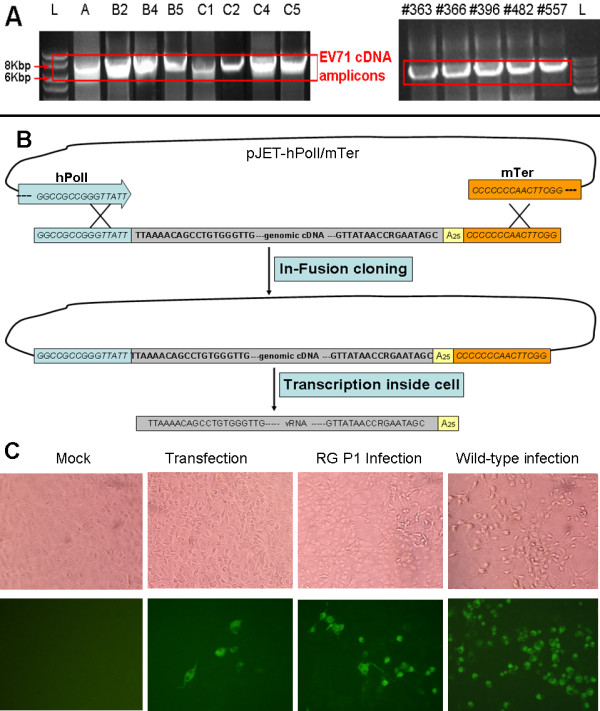
**Rescue of EV71 viruses by hPolI-driven reverse genetics (RG) system.** (**A**) Amplification of the full-length genomic cDNA of EV71 (~ 7.5 Kb). RNA of wild-type EV71 strains belonging to subgenogroup A, B2, B4, B5, C1, C2, C4 or C5 and 5 unknown EV71 strains (#363 to #577) from Malaysia was extracted and amplified using two universal primers EV71-Uni-F and EV71-Uni-R in a fast RT-PCR reaction. L represented 2log DNA ladder (Bio-rad). (**B**) Strategy for the construction of EV71 RG plasmid. The linear pJET-hPolI/mTer vector and EV71 cDNA amplicon had 15 identical nucleotides at both 5^′^ and 3^′^ ends. They were joined together so that EV71 cDNA was directly flanked by hPolI promoter and murine terminator (mTer) using In-Fusion cloning method. The recombinant plasmid produced authentic and infectious viral genomic RNA upon transfection into Vero cell. (**C**) CPE and IFA identification of the rescued EV71-B5 RG virus. Vero cells infected by the EV71-B5 RG or wild-type viruses were observed for CPE at 5 days after infection. The mock and pJET-hPolI/mTer-EV71-B5 plasmid transfected cells did not show CPE. IFA signals were detected at 24 h in the tranfected cells and cells infected with RG or wild-type B5 virus; while mock cells were negative after treating with guinea pig anti EV71 serum.

Initially, two primers EV71-Uni-F and EV71-Uni-R were designed to take advantage of the conserved viral RNA termini and contain 15 extra bases at the 5^′^ end for joining with hPolI promoter and murine terminator (mTer) during the In-Fusion reaction. The genomic RNAs of EV71 strains of different subgenogroups (A, B2, B4, B5, C1, C2, C4 or C5) isolated from 1970 to 2010 were efficiently amplified by RT-PCR using above primers. Moreover, to help combat and study the emerging EV71 strains from Malaysia, the unknown genomes of 5 strains #363 to #577 (Figure 1A), isolated in 2010, were amplified, sequenced and genotyped. Although all the strains belonged to subgenogroup B5, new mutations, especially in the VP1 gene, were discovered (data not shown). The EV71 cDNA amplicons had a size of about 7.5 kb and were clearly visible on the agarose gels stained with ethidium bromide (Figure 1A). The purified cDNA amplicons were directly joined with linearized pJET-hPolI/mTer vector during the In-Fusion cloning reaction without any enzyme digestion (Figure 1B). During the PCR screening next day, more than 80% of the colonies showed positive recombinations (data not shown), which indicated that this method is reliable.

To determine whether RG viruses were rescued and infectious after directly transfection of recombinant plasmids, both transfected and RG virus infected cells were analyzed by CPE and IFA. Here, only EV71-B5 was presented as an example because other EV71 strains showed a similar pattern. After the transfection of the recombinant plasmid pJET-hPolI/mTer-EV71-B5, we did not observed the CPE under light microscopy at the third post transfection day because little RG viruses were generated from EV71-B5 cDNA constructs. However, the CPE was obvious when fresh Vero cells were infected by rescued RG EV71-B5 from transfection (P1 infection, Figure 1C). Furthermore, IFA results showed that transfected or RG virus infected cells were positive by immunostaining with guinea pig anti EV71 serum (Figure 1C). Additionally, the viral proteins VP1 and 3D were both detectable after the transfection and infection using mouse monoclonal antibodies 1D9 and 4B12 (Additional file 1). Therefore, the hPolI promoter was active and the EV71 genomic RNA was successfully transcribed inside Vero cell, and the rescued RG EV71-B5 viruses were infectious and produced viral proteins inside cells.

**Figure 2:**
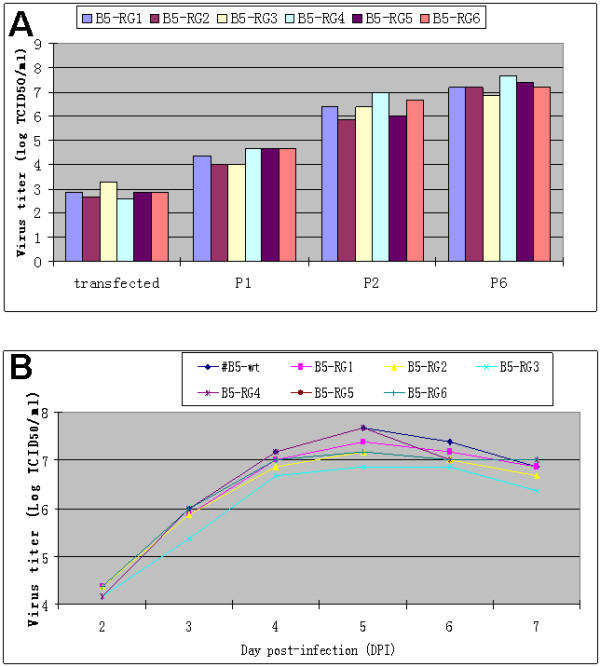
**Comparative study of growth of EV71-B5 RG and wild-type (wt) viruses.** (**A**) Growth titer (TCID_50_) of six rescued EV71-B5 RG viruses at different passages in Vero cells. Each column represents the arithmetic mean value of two duplicated experiments. (**B**) One-step growth curves of three selected B5 RG viruses and the wild-type B5 in Vero cells. The cells infected at a MOI of 1 were harvested at several time points and the virus titer (TCID_50_) was determined. The data shown are the arithmetic mean values of duplicated experiments.

To further compare the growth characteristics of the rescued virus and wild-type virus, their growth titers at different passages and a one-step growth curve were studied. Six positive recombinant plasmids pJET-hPolI/mTer-EV71-B5 were separately transfected into Vero cells. After 3 days, 1ug of each plasmid produced around 10^3^ TICD_50_ infectious viruses (Figure 2A). After the first and second passage, the RG virus titers grew to above10^4^ and 10^6^ TICD_50_ /ml in Vero cell, respectively; and the titer was high enough to establish a virus seed stock. At the sixth passage, the growth titer of RG viruses was just slightly lower than wild type viruses (Figure 2A), which indicated the RG virus is suitable to be grown at large scale for vaccine production. The growth kinetics of the six RG viruses (B5-RG1 to B5-RG6) from the 5^th^ passage in Vero cell was examined by one-step growth curve study. The results showed that the rescued B5-RG viruses displayed as statistically similar titers at each time point as the wild type B5 in Vero cell. Peak titers were observed at the day 5 post-infection for both wild type and RG viruses (Figure 2B).

To test whether the RG viruses retained the same immunogenicity as wild-type virus, two RG viruses B5-RG1 and B5-RG2 were randomly chosen for immunogenic study in mice. The formalin inactivated whole EV71-B5 RG and wild type viruses with AlPO_4_ adjuvant were injected into mice on day 0 and 14. Mouse immunogenicity studies (Table 1) revealed that two RG viruses B5-RG1 and B5-RG2 were immunogenic and elicited good cross-genogroup virus neutralization titers (2^8^ for homologous B5 strain and > 2^4^ for other heterologous EV71 strains) on day 21. In addition, the neutralization titers elicited by RG and wild-type of EV71-B5 were statistically identical. The results indicated that the RG viruses retained the immunogenicity of their original wild-type virus. Hence, the pure viruses rescued by this novel RG system have great potential advantages in vaccine production.

**Table 1:** Comparison of immunogenicity of formalin-inactivated RG and wild-type (wt) viruses

**Antigen**	**Log2 (titer of neutralizing antibody) against (mean (SD)):**						
	**EV71-B2**	**EV71-B4**	**EV71-B5**	**EV71-C1**	**EV71-C2**	**EV71-C4**	**EV71-C5**
B5-wt	7(0.89)	7.8(0.98)	8(0.89)	5.1(0.75)	5.7(0.75)	4.2(0.75)	4.7(0.82)
B5-RG1	7(1.26)	7.3(1.03)	7.8(0.75)	4.8(0.75)	5(0.63)	4.3(0.82)	4.83(0.75)
B5-RG2	7(0.89)	7.16(1.17)	8 (0.63)	4.6(0.82)	5.2 (0.4)	4.2(0.98)	4.5(0.55)
PBS	< 3	< 3	< 3	< 3	< 3	< 3	< 3

Cross-neutralization antibodies titers elicited in mice by RG and wt EV71-B5 viruses were almost the same against homologous or heterologous EV71 strains from different subgenogroups. The experiment was duplicated and the data represents the arithmetic mean value (n=6)±SD.

## Discussion

**Figure 3:**
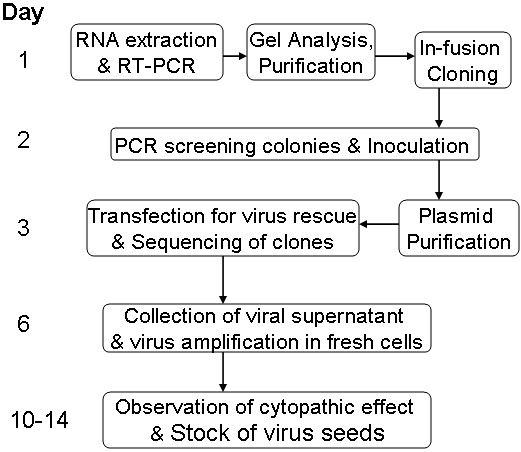
**Illustration of a streamlined scheme for rapid production of EV71 RG viruses that could be used as vaccine seed stocks.** The illustration begins with a clinical EV71 isolate which could be from any subgenogroup.

In conventional studies, the infectious clones of single-stranded positive RNA viruses with large genome, such as poliovirus and FMDV, were constructed by elaborate ligation of several subgenomic clones generated from multiple, segmented RT-PCR due to the difficulties in long template PCR amplification [19-21]. To amplify the full length 7.5 kb cDNA of EV71 genome, we designed a pair of universal primers which could successfully amplify all known EV71 strains in a fast PCR reaction using high fidelity pfuUltra DNA polymerase. Because of genetic diversity of EV71, the selection of restriction enzymes for plasmid construction was laborious. In our system, the cDNA amplicons were directly joined with the linearized vector in the In-fusion cloning reaction. The whole procedure could be done in only one day (Figure 3).

In our previous paper, we described an hPolI-driven RG system for generating infectious EV71 from synthetic cDNA in RD cell [18]. In this study, we tried a similar system to rescue EV71 viruses in Vero cell for vaccine production. This RG system takes advantage of host RNA polymerase I promoter to generate exact EV71-like vRNA with precise initiation and termination inside Vero cell. Unlike to T7 or SP6 polymerase-driven RG systems, no extra bases were added during *in vivo* transcription at the 5^′^ and 3^′^ ends of viral RNA transcripts (Figure 1B). Those extra bases had been reported to impede the rescue of some RG viruses [22,23].

Traditionally, potential EV71 vaccine strains are firstly selected from hundreds of clinical isolates which are usually cultured in RD cell. Candidate strains with low virulence and high immunogenicity are then attenuated in Vero cell which has been approved for EV71 vaccine production [13,24]. Plaque screening and further subculture are then performed to select and purify the candidate strains with higher growth titers. The genomic stability and immunogenicity of candidate strains in passages have to be maintained. The whole procedure is time-consuming and haphazard, and contamination with pathogens derived from the clinical samples or non-validated cell culture systems can occur [25]. In contrast, our hPolI-driven RG system our procedure was faster because it excludes the chance of contamination by other pathogens resulting in pure virus stock from an identical cDNA construct in 10 days (Figure 3). Additionally, this method is less haphazard because the exact genome sequence for each clone was known from the beginning which permits genomic comparison and engineering to exclude clones carrying virulence determinants or to introduce desired traits.

## Conclusions

In conclusion, we have established a robust RT-PCR method to amplify the entire genome of EV71, regardless of its subgenogroup, with a pair of universal primers, and used these amplicons for both high-throughput sequencing and direct incorporation into a reverse-genetics plasmid containing an hPolI promoter to rapidly produce infectious EV71 seed stock. The whole procedure took 10 days only. Furthermore, the rescued RG viruses retained the same immunogenicity as wild type viruses in mouse. This RG system greatly simplifies the isolation and selection of EV71 vaccine candidates and could accelerate the development of EV71 vaccines.

## Materials and methods

### Cell culture and virus propagation

Human rhabdomyosarcoma (RD) cell and Vero cell were maintained in Minimum Essential medium (MEM, Gibco) containing 10% fetal bovine serum (FBS, iDNA) at 37°C in the presence of 5% CO_2_. The EV71 strains used in this study were EV71-A (BrCr, U22521.1), EV71-B2 (7432/MS/87, U22522.1), EV71-B4 (HFMD41 /SIN), EV71-B5 (NUH0083/SIN/08, FJ461781), EV71-C1 (Y90-3761, AB433864), EV71-C2 (1585-Yamagata-01, AB177812), EV71-C4 (75-Yamagata-03, AB177813), EV71-C5 (3437/SIN/06, GU222654), and some unknown EV71 strains from Malaysia labeled #363, #366, #396, #482, and #557. All EV71 wild type strains were isolated from patients and propagated in RD cell. All reverse genetics viruses were rescued in Vero cells.

### EV71 genomic cDNA amplification

Viral RNA was extracted from culture supernatant using RNeasy mini kit (Qiagen). The reverse transcription reaction was performed for 1 hour at 42°C with RevertAid First Strand cDNA Synthesis Kit (Fermentas) using viral RNA as the template and oligo(dT)_18_ as primer. The full-length genomic cDNAs of EV71 were amplified using PfuUltra II Hotstart PCR Master Mix (Agilent Technologies, Inc) and EV71 specific universal primers EV71-Uni-F (5^′^-GGCCGCCGGGTTATTTTAAAACAGCCTGTGGGTTG) and EV71-Uni-R (5^′^-CCGAAGTTGGGGGGGTTTTTTTTTTTTTTTTTTTTTTTTTTGCTATTCYGGTTATAAC). The reaction parameters were as follows: an initiation step at 94°C for 2 min, followed by 40 cycles (94°C for 15 sec, 52°C for 20 sec and 72°C for 4 min) and a final extension step at 72°C for 5 min. The amplicons were analyzed by gel electrophoresis and purified using QIAquick gel Purification Kit (Qiagen).

### Construction of recombinant plasmids for RG EV71

The human RNA polymerase I (hPolI) promoter and murine terminator cassette sequence were amplified from sap/pol vector and then inserted into pJET1.2 vector as described previously [18]. The plasmid pJET-hPolI/mTer was linearized by PCR using primers pJET-HPol1mTer-f (5^′^-CCCCCCCAACTTCGGAGGTC) and pJET-hPol1mTer-r (5^′^- AATAACCCGGCGGCCCAAAATG). The EV71 genomic cDNA amplicons were joined with linearized pJET-hPolI/mTer using In-Fusion HD cloning Kit (Agilent Technologies, Inc). 5 μl of the cloning reaction was transformed into 100 μl of competent XL1-Blue E. coli by heat shock. The recombinant plasmids were selected by PCR screening with primers pJET-f (5^′^-CGACTCACTATAGGGAGAGCGGC) and EV71-VP1-r (5^′^-GCYCCRTATTC AAGRTCTTTCTC), and their correctness and genotypes were confirmed by DNA sequencing and phylogenetic tree analysis, respectively.

### Transfection and infection

A mixture of 1 μg of each recombinant plasmid and 2.5 μl Lipofectamine 2000 reagent (Invitrogen) was transfected into 5x10^5^ Vero cells in 2 ml medium in 6-well plates. The transfected cells were lysed after 3 days by 3 freeze-thaw cycles and the supernatant containing viruses was collected for further passage. The rescued viruses were harvested after 4 days and their titers were determined as 50% tissue culture infective dose (TCID_50_) according to cytopathic effect (CPE) in Vero cell using the Reed and Muench formula.

### One-step growth curve of rescued viruses

The growth kinetics of rescued viruses was determined in subconfluent Vero cell culture in 1 ml medium in 24-well plates. The cells were incubated with rescued viruses at a multiplicity of infection (MOI) of 1 for 1 h at 37°C, and then washed with PBS 3 times and cultured in MEM with 2% FBS. The infected cells were frozen at day 2, 3, 4, 5, 6 and 7 post-infection and stored at -80°C. After 3 freeze-thaw cycles, the virus concentrations were titrated by TCID_50_ assay.

### Indirect immunofluorescence assay (IFA)

Vero cells transfected with the recombinant plasmids or infected with rescued RG virus were grown in 96-well plates. The cells were fixed with 4% PFA after 24 h and incubated with guinea pig anti EV71 serum (produced in house) diluted 1:500 for 30 min at 37°C. Then the cells were washed and incubated with fluorescein isothiocyanate (FITC)-labeled goat anti-guinea pig IgG (Dako) diluted 1:200 for another 30 min. Finally, the cells were rinsed with PBS and visualized under a fluorescent microscope (Olympus).

### Immunogenicity of RG viruses in mouse

The rescued and wild type viruses in Vero cell were harvested at 5^th^ day post-infection and purified by ultracentrifugation as described previously [18]. The purified viruses were then inactivated with 0.2% formalin (v/v) at 37°C for 3 days and stored at 4°C, and the amount of viral protein was determined using Bradford assay (Bio-Rad Laboratories, USA). For immunization, groups of 6 female BALB/c mice (6 to 8-week old) were each intraperitoneally immunized with 25 μg inactivated viruses in 200 μl of 0.1 M aluminum phosphate (Sigma) or PBS as a negative control. The mice were boosted with the same dose 2 weeks after priming. The sera were then collected one week after the boost and the presence of neutralizing antibodies against EV71 was assayed by an *in vitro* microneutralization assay [18]. Briefly, 25 μl of serial twofold dilutions of heat inactivated serum samples were mixed with 25 μl of 100 TCID_50_ of virus, and the neutralization antibody titer was determined as the highest dilution of serum that protected 50% of Vero cell cultures.

All animal experiments were carried out in accordance with the Guides for Animal Experiments of the National Institute of Infectious Diseases (NIID), and experimental protocols were reviewed and approved by the Institutional Animal Care and Use Committee of the Temasek Life Sciences Laboratory, Singapore (Project Approval no. TLL-11-002).

## Abbreviations

EV71: Enterovirus 71; HFMD: Hand foot and mouth disease; ORF: Open reading frame; UTR: Untranslated region; hPolI: Human RNA polymerase I; mTer: Murine terminator; RG: Reverse genetics; CPE: Cytopathic effect; IFA: Immunofluorescent assay; MOI: Multiplicity of infection; TCID_50_: 50% tissue culture infection dose; MEM: Minimum essential medium; Vero: African green monkey kidney cell.

## Competing interests

The authors declare that they have no competing interests.

## Authors’ contributions

TM performed the experiments. TM and TKK drafted the manuscript. TM and JK designed the study. JK supervised the work and edited the final version of this manuscript. All authors read and approved the final manuscript.

## Supplementary Material

Additional file 1 **(C) IFA identification of the rescued EV71-B5 RG viruses.** The viral proteins were detected with mouse monoclonal antibodies 1D9 and 4B12 which were raised against VP1 and 3D of EV71, respectively. The pJET-hPolI/mTer-EV71-B5 plasmid transfected cells and RG EV71-B5 or wild-type B5 infected cells showed positive IFA signals at 24 h; while mock cells were negative. Click here for file
