# Supplementary figures and images for: RNA polymerase I-driven reverse genetics system for enterovirus 71 and its implications for vaccine production
Source: Virol J. 2012 Oct 17;9:238. doi: 10.1186/1743-422X-9-238 (PMC3493273; doi:10.1186/1743-422X-9-238)

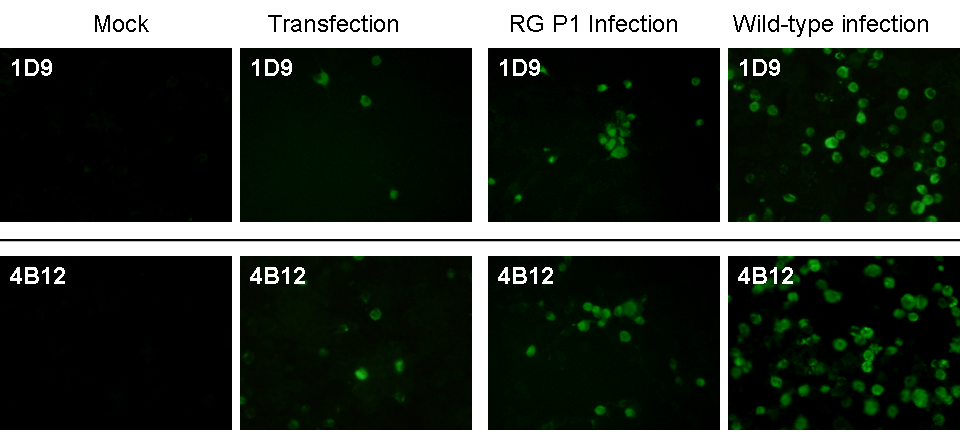

Supplement: Additional file 1 — (C) IFA identification of the rescued EV71-B5 RG viruses. The viral proteins were detected with mouse monoclonal antibodies 1D9 and 4B12 which were raised against VP1 and 3D of EV71, respectively. The pJET-hPolI/mTer-EV71-B5 plasmid transfected cells and RG EV71-B5 or wild-type B5 infected cells showed positive IFA signals at 24 h; while mock cells were negative. [file 1743-422X-9-238-S1.tiff]
